# Oxidative Stress Responses in Obese Individuals Undergoing Bariatric Surgery: Impact on Carcinogenesis

**DOI:** 10.3390/pathophysiology31030026

**Published:** 2024-07-04

**Authors:** Daniel Araki Ribeiro, Glenda Nicioli da Silva, Ingra Tais Malacarne, Luciana Pellegrini Pisani, Daisy Maria Favero Salvadori

**Affiliations:** 1 Department of Biosciences, Federal University of Sao Paulo—UNIFESP, Santos 11015-020, SP, Brazil; ingra_tais@hotmail.com (I.T.M.); pisani@unifesp.br (L.P.P.); 2 Department of Clinical Analysis, Federal University of Ouro Preto—UFOP, Ouro Preto 35402-163, MG, Brazil; nicioli@ufop.edu.br; 3 Department of Pathology, Botucatu Medical School, Sao Paulo State University—UNESP, Botucatu 18618-687, SP, Brazil

**Keywords:** gastroplasty, oxidative stress, genotoxicity, cytotoxicity, cancer risk

## Abstract

Obesity is a big public health problem that claims several thousand lives every year. Bariatric surgery has arisen as a suitable procedure for treating obesity, particularly morbid obesity. Oxidative stress, genotoxicity, apoptosis, and inflammatory responses are recognized as the most important occurrences in carcinogenesis, as they actively contribute to the multistep process. This study aimed to briefly review the connection between oxidative stress, genotoxicity, apoptosis, and inflammation in obese patients undergoing bariatric surgery, focusing on its impact on carcinogenesis. Regarding oxidative stress, bariatric surgery may inhibit the synthesis of reactive oxygen species. Moreover, a significant reduction in the inflammatory status after weight loss surgery was not observed. Bariatric surgery prevents apoptosis in several tissues, but the maintenance of low body weight for long periods is mandatory for mitigating DNA damage. In conclusion, the association between bariatric surgery and cancer risk is still premature. However, further studies are yet needed to elucidate the real association between bariatric surgery and a reduced risk of cancer.

## 1. Introduction

Obesity is a significant public health problem, contributing to several thousand deaths every year. According to data released by the World Health Organization, over 600 million people worldwide have obesity [1]. A growing body of evidence demonstrates that obese individuals constitute a high-risk group for immediate and late complications. Obesity is closely related to other chronic disorders (diabetes mellitus, hypertension, steatosis, and cardiovascular disease) [2]. Several therapies have been suggested for the treatment of obesity, including lifestyle interventions, anti-obesity medicines, and surgery. The four drugs approved by the U.S. Food and Drug Administration (orlistat and liraglutide, and two combination drugs, topiramate-phentermine and bupropion-on-naltrexone) are not as effective as surgery, adding an average of about 5% to the weight loss of one’s lifestyle. This was significantly improved by two new drugs under investigation, semaglutide and tirzepatide. However, there is considerable variability in the amount of weight lost with these medications, as there is with diet, surgery, and lifestyle. Individuals can benefit from a well-designed lifestyle program by losing an average of 8%, and in some cases, exceeding 15% of their body weight. However, there are an equal number of people who experience little or no weight loss benefit from this intensive therapy [3]. Despite lifestyle intervention efficacy, bariatric surgery has been described as the best therapeutic option for sustained weight loss, especially in advanced cases, i.e., morbid obesity and related comorbidities [4]. It has been demonstrated that weight loss in obese individuals reduces low-density lipoprotein cholesterol (LDL-C) levels, triglycerides, soluble intercellular adhesion molecule 1, fasting glucose concentrations, and systolic and diastolic blood pressure [5]. In fact, multiple follow-up studies have assumed successful control of diabetes mellitus and hypertension in association with weight loss from bariatric surgery, as well as a significant reduction in mortality [6,7,8,9]. Moreover, several researchers have documented that bariatric surgery is able to decrease the cancer risk in these individuals [10,11].

Regarding clinical investigation, some epidemiological studies have assumed a positive association between visceral adiposity and the incidence of neoplasia [12,13]. This ratifies the logical rationale that obesity is not only a risk factor for cancer but also plays a key role in its etiopathogenesis. Several factors have been advocated, such as the connection between adipocytes and inflammatory cells by means of cytokines and reactive oxygen species synthesis; the endocrine signaling pathways of adipocytes, both through action of androgens and gastrointestinal hormones, such as resistin, leptin, and visfatin; the meaning of the microbiome and its modifications in the scenario of obesity; the outcomes of insulin resistance and diabetes on cancer cell survival; individual genetic predisposition and/or epigenetic changes, etc. [8]. Taken as a whole, the role of adipose tissue in this biological context plays a crucial role in inflammation, tissue fibrosis, and extracellular matrix remodeling; in the microenvironment that interferes with lipid metabolism and causes insulin resistance; in microbiota dysbiosis and impaired immune function; and in imbalanced sex hormones and adipokine secretion [9,10]. However, the risk of cancer in patients with obesity undergoing bariatric surgery is controversial so far. In a recent review published by Lim et al. [13], bariatric surgery was associated with a low incidence of developing breast and gynecological cancers (endometrial and ovarian cancers) [13]. Conversely, the relationship between bariatric surgery and the development of gastrointestinal cancers (gastric, esophageal, liver, and pancreas) is highly heterogeneous, with studies showing either no association or even a low incidence [13]. Moreover, some preclinical and cohort studies assumed an increased risk of developing colon and rectal cancer after bariatric surgery [13]. It is important to stress that studies evaluating the positive or negative effects of weight loss after bariatric surgery were only conducted 1 or 2 years after surgery. What happens next in terms of cancer risk is no longer necessarily related to the direct results of bariatric surgery but to the impact of biological pathways on a new clinical scenario. For this reason, it is very important to elucidate such events closely related to oxidative stress responses for a better understanding of the outcomes induced by bariatric surgery in these patients.

Specifically, Mengoni [14] discussed body composition measurements as biomarkers for melanoma in patients who were treated with tyrosine kinase inhibitors (TKIs). The authors have proposed a putative use of the visceral adipose tissue gauge index (VATGI) as a biomarker for predicting patient outcome. It was discussed that adipose tissue and skeletal muscle also serve as active endocrine organs, releasing so-called adipokines and myokines that may influence treatment responses. Furthermore, progressive sarcopenia and myosteatosis are associated with a poor clinical outcome in patients with advanced hepatocellular carcinoma (HCC), and high baseline visceral adiposity is related to worse survival [15]. In addition, Xu et al. [16] have postulated that visceral fat distribution is more predictive of the clinical risk of primary liver cancer (PLC) than common in vitro measures, discussing that liver fat deposition leads to non-alcoholic fatty liver disease (NAFLD), non-alcoholic steatohepatitis (NASH), and cirrhosis, eventually leading to PLC. Moreover, lipid-depleted visceral adipose tissue was associated with advanced tumor characteristics in non-metastatic clear cell renal cell cancer (ccRCC). In ccRCC, the tumor microenvironment consists of visceral adipose tissue surrounding the kidney (perinephric fat) in the advanced stages of the disease [17]. Finally, Popovici [18] showed that overweight is a significant predictive factor for rectal cancer, discussing that obesity is most likely related to the activation of myeloperoxidase-positive cells within the colorectal mucosa, assuming an underlying inflammatory response.

Carcinogenesis involves a sequence of events that transform normal cells into malignant cells. The important and mandatory events for carcinogenesis are initiation, promotion, and progression. During the initiation phase, physical, chemical, or biological agents induce genetic damage (genotoxicity). Subsequently, the damage becomes permanently incorporated into the genetic apparatus if DNA breakage is not repaired properly [19]. In the promotion phase, many mutated cells proliferate in a disorganized way [20]. The multiplication of these cells contributes to tumor formation (progression phase). It is important to emphasize that genotoxicity plays a role in all steps of carcinogenesis, particularly during the initiation stage.

Oxidative stress leads to the activation of several biological pathways that culminate in an oxidative imbalance that damages cells and tissues, thereby contributing to carcinogenesis [21]. Reactive oxygen species (ROS) generation, partially reduced metabolites of oxygen with strong oxidizing properties, may be caused by endogenous origin as depicted by hepatic function and subsequent action of the enzyme P450, mitochondrial activities, or exogenous factors, such as chemical compounds [22]. ROS may participate in carcinogenesis by causing mutations in genes that are closely involved in proliferative activity and an imbalance in transduction and/or transcription signals [22]. In addition, the increased metabolism of cancer cells is due to the upregulation of glucose transport, which is independent of oxygen supply. The Warburg effect increases the steady-state ROS state in cancer cells by lactate synthesis, which is extruded by monocarboxylate transporters in cancer cells, and the lack of antioxidant properties, unlike pyruvate, citrate, malate, and ox-aloacetate, along with reducing equivalents (NADH/H+), which are antioxidant metabolites [23]. In light of these considerations, the hyperlipidemic state of obesity leads to an increase in free fatty acids (FFAs), causing excess ROS [24]. Additionally, ROS are increased in adipocytes since FFAs can activate NADPH oxidase, an enzyme closely involved in superoxide radical synthesis [25].

The close relationship between inflammation and cancer is well established in the development of neoplasms [26,27]. Inflammatory cells and pro-inflammatory cytokines are frequently present in tumors, which is an important biological scenario for developing neoplasia through oxidative DNA damage and subsequent impairment of the DNA repair system [28]. Leukocytes impair the redox system and generate genomic instability [28].

A previous study has shown that inflammatory activity induced by leukocyte activation is associated with low levels of nitric oxide [29]. For example, macrophages from cocaine users are unable to utilize nitric oxide as an antibacterial molecule [30]. The role of nitric oxide in contact allergen-induced skin inflammation is also complex. At low concentrations, nitric oxide is pro-inflammatory by inducing vasodilation and neutrophil recruitment, while at high concentrations, it down-regulates adhesion molecules, suppresses activation, and induces the apoptosis of the inflammatory cells [31]. Taken as a whole, a positive association between carcinogenesis, oxidative stress, genotoxicity, apoptosis, and obesity-induced inflammatory responses may be established. For this reason, the clinical relationship between obesity and cancer has been revealed in recent decades [32,33]. However, to the best of our knowledge, it is not established the impact of weight loss after bariatric surgery on cancer incidence. Particularly, it is yet unclear if there is a putative relationship between drastic weight loss after bariatric surgery and cancer risk in the postoperative period [34]. Certainly, investigating the role of oxidative stress, genotoxicity, apoptosis, and the inflammatory response in obese individuals undergoing bariatric surgery will elucidate the issue. Therefore, the aim of this review was to search for scientific data published in the field for a better understanding of these associations.

## 2. Search Strategy

A critical literature search for studies on obesity, bariatric surgery, DNA damage (genotoxicity), inflammation, and oxidative stress was performed. In brief, a search of PUBMED/Medline, Scopus, and Web of Science for a variety of articles (all publications until July 2024) was carried out. Case reports and papers not written in English were excluded from the study. All papers were critically ready by the authors and included in this review.

## 3. Oxidative Stress

Obesity may impair the redox balance in mammalian cells, resulting in oxidative stress. Consequently, obese individuals develop severe oxidative stress in several tissues and organs. Some authors have also demonstrated elevated oxidative stress in the peripheral blood lymphocytes of severely obese individuals [35]. Since high oxidative stress is closely associated with morbidity and mortality risks, such as cancer, it is crucial to understand the etiopathogenesis of obesity and related diseases.

Over the last few decades, bariatric surgery has emerged as the most effective treatment for morbid obesity. Understanding the pathobiological pathways involved in oxidative stress in individuals undergoing bariatric surgery is an important strategy for elucidating the precise outcomes induced by surgical intervention, particularly the impact of oxidative stress on various tissues and organs. Two previously published clinical investigations have reported that bariatric surgery causes weight loss under oxidative stress. Twenty-four months after bariatric surgery, a relevant decline in insulin resistance, leptin levels, inflammation, and oxidative stress was observed [36]. In addition, oxidative stress was reduced in 16 obese individuals at 6 months after bariatric surgery [37]. Recently, a decrease in the levels of oxidized low-density lipoprotein followed by an increase in the antioxidant enzymes paraoxonase-1 and catalase one year after bariatric surgery has been documented [38]. Furthermore, total antioxidant activity increased four years after bariatric surgery (Roux-en-Y gastric bypass, sleeve gastrectomy, biliopancreatic diversion, and laparoscopic adjustable gastric banding), although the TBARS (thiobarbituric acid reactive substances) assay in plasma showed no significant changes [39]. However, it has also been argued that post-bariatric surgery-related hypoglycemia is a physiological situation capable of inducing oxidative stress in human blood cells [40,41]. These findings were confirmed by increasing HDL-cholesterol levels, followed by no remarkable changes to triglycerides or LDL-cholesterol levels [42]. Moreover, antioxidant supplementation may be explored as a therapeutic option to decrease oxidative stress in individuals undergoing bariatric treatment [43]. This opens up the testing of new biomolecules that could interfere with the production of free radicals and lead to the reversal or even retardation of diseases caused by oxidative and inflammatory processes, such as obesity.

A prospective study demonstrated that individuals who underwent bariatric surgery reduced DNA damage as a result of improving sperm mitochondrial activity, but the procedure was able to decrease lipid peroxidation levels after 12 months in seminal cells [44]. When the results of two surgical procedures (Roux-en-Y gastric bypass and sleeve gastrectomy) were compared, the Roux-en-Y gastric bypass group showed a significant decrease in plasma serum TBARS, which are advanced oxidation protein products. In the sleeve gastrectomy group, the levels of advanced oxidation protein products were related to serum C-reactive protein levels [45]. In addition, urinary excretion of 8-oxodG was elevated in the first month after Roux-en-Y gastric bypass. Two years after Roux-en-Y gastric bypass, a reduction in 8-oxoGuo levels was detected. Interestingly, the excretion levels were ~30% lower in the female subgroup for both markers evaluated [46]. Several oxidative biomarkers were measured in saliva, and the results showed that advanced oxidation protein products, protein carbonyl groups, and DNA 8-hydroxy-D-guanosine (8-OHdG) were significantly higher in morbidly obese individuals than in those who had undergone bariatric surgery six months earlier [47]. Overall, Roux-en-Y gastric bypass appears to be more effective in reducing oxidative damage following surgical treatment [48].

Currently, knowledge of the biological mechanisms underlying the cardioprotective benefits of bariatric surgery remains limited. Abad-Jimenez et al. [49] investigated whether weight loss was induced by Roux-en-Y gastric bypass as a result of improving the oxidative status of leukocytes. The results demonstrated that Roux-en-Y gastric bypass improved some metabolic indices (glucose and lipid levels) and oxidative stress, as superoxide generation and protein carbonylation were reduced [49]. It is plausible to assume that Roux-en-Y gastric bypass improves the pro-oxidant status of leukocytes in individuals [49]. When obese people undergoing laparoscopic sleeve gastrectomy were evaluated, similar results were found, i.e., the oxidative stress status was significantly improved [50,51,52].

A fascinating alternative approach for reducing oxidative stress is to inhibit ROS production by blocking the enzymes involved in its synthesis, but most of the articles cited here include small sample sizes and larger population studies in order to confirm the results.

## 4. Inflammatory Response

It has been shown that O_2_ generates H_2_O_2_ after reacting with manganese SOD (MnSOD) in the mitochondria, activating redox-sensitive transcription factors (HIF-1α and NFκB) and pro-inflammatory cytokines. This biological scenario activates inflammatory caspases (caspase-1 and -12) and cytokines (IL-1β and IL-18) in macrophages. Inflammatory cells synthesize ROS by activating oxidant-generating enzymes such as, for example, NADPH oxidase, iNOS, xanthine oxidase (XO), and myeloperoxidase (MPO), which contribute to the mutational burden through damage to DNA, RNA, lipids, and proteins. Inflammatory tissues also release some cytokines able to activate NFκB, which activates COX2, lipoxygenase (LOX), and iNOS, leading to excessive production of ROS. Inflammation promotes the development and progression of cancer by means of vascularization and remodeling some tumor components, which is crucial for cell survival and tumor growth [23].

Owing to the relevance of the immune system in obese individuals, it is interesting to assess whether there is an inflammatory response in adipose tissue after bariatric surgery. In morbidly obese individuals, an association between inflammatory markers and weight loss through laparoscopic adjustable gastric banding surgery was observed; C-reactive protein (CRP) levels were elevated pre-surgery; however, 18 months after surgery, CRP levels showed a significant reduction [53]. Another group of studies analyzed people who underwent bariatric surgery for Roux-en-Y gastric bypass surgery at four time points: immediately after the intervention, at 3 weeks, and at 3 and 6 months [54]. The concentration of leptin decreased by 70% 6 months after surgery, and adiponectin did not show significant changes at any time points [54]. Such findings suggest that leptin is closely involved in weight loss. However, it is important to stress that other conditions are also involved in leptin secretion, such as, for example, nutrient intake, energy-metabolizing hormones, eicosanoids, and pathological conditions [54]. Recently, another study analyzed the inflammatory behavior in a group of obese individuals diagnosed with type 2 diabetes mellitus and another group of obese individuals without the disease after Roux-en-Y gastric bypass surgery and compared it with that of healthy controls [55]. First, the levels of IFN-β, IL-6, and IL-18 were higher in both groups of obesity when compared to the control, and only the levels of TNF-α, IL-15, IL-1α, and IL-2 were increased in obese individuals who did not have diabetes when compared to the control group [54]. Six months after the surgical intervention, only IL-6 levels were increased in both obese groups, while non-diabetic individuals had increased levels of TNF-α, IL-15, and IL-18, and obese diabetic individuals showed changes in IL-27 levels [54]. Interestingly, only two cytokines showed variability in their plasma concentrations among initially obese individuals; however, the increase in TNF-α levels was correlated with body mass index (BMI) and age, and IL-2 levels decreased with respect to age in obese non-diabetic individuals. No differences were found between the obese groups when a correction analysis for multiple tests was performed. Importantly, after the surgical intervention, there was an increase in inflammation in obese diabetic individuals; however, in non-diabetic individuals, the inflammation present in the initial stages after surgery was maintained for the next six months [54].

A study that evaluated women undergoing gastric bypass surgery at three time points (after 3, 6, and 12 months) observed that 12 months after the surgery, IL-6 and hs-CRP had lower concentrations when compared to pre-surgical data [55]. On the other hand, adiponectin was present at higher concentrations, and curiously, TNF-α showed transient results in its analysis, being increased but without statistical significance. Surprisingly, when the levels of the inflammation markers were correlated with BMI, HOMA, and insulin 12 months after the intervention, only IL-6 and hs-CRP were found to be positively associated [55]. Another study evaluated the presence of inflammation preoperatively and three months later in patients undergoing bariatric surgery (sleeve gastrectomy or Roux-en-Y gastric bypass), patients with lipodystrophy, and a control group [56]. The inflammation markers that showed an increase in their plasma concentrations were IL-10, ANXA-1, and adiponectin; CRP and leptin showed reductions three months after surgery, showing the presence of mild systemic inflammation after three months [56]. Moreover, an increase in adiponectin and IL-10 expression was observed in a morbidly obese population one year after performing the surgical procedure (sleeve gastrectomy or Roux-en-Y gastric bypass) [57]. Conversely, the levels of IL-6, leptin, and resistin decreased [49]. Interestingly, when a multivariate analysis correlating the decrease in these inflammatory markers following weight loss was performed, only IL-10 did not show a positive correlation [57]. Therefore, most inflammatory markers showed an increase in their post-surgery levels; however, IL-6 showed a variable trend in the results, and in most studies, it was lower. The decrease in post-operative leptin, resistin, hs-CRP, and CRP levels and the increase in levels of IL-10, anexin-1 (ANXA-1), adiponectin, TNF-α, IL-5, and IL-18 after metabolic surgeries suggest that these markers may be useful for detecting a chronic inflammatory process after metabolic surgery, regardless of the surgical technique used.

When the inflammatory behavior in an obese population after a surgical procedure for weight loss (vertical sleeve gastrectomy, VSG, or Roux-en-Y gastric bypass) was investigated, leukocytes pointed out a significant increase in adipose tissue after 1 month compared to baseline, and there was also a glucose tolerance improvement [58]. Nevertheless, no differences were found in the levels of other biomarkers in the same tissue or in the levels of circulating inflammatory markers, such as IL-6 [58]. Taken together, these data are consistent with the hypothesis that despite metabolic improvements, there is no reduction in the inflammatory condition, even after weight loss surgery [59]. Another study compared the plasma levels of IL-6 and TNF-α in morbidly obese individuals after laparoscopic bariatric surgery and non-morbidly obese control individuals who underwent laparoscopic gastrectomy for early gastric cancer or benign gastric tumors [59]. Plasma IL-6 levels were lower in morbidly obese individuals after the surgical intervention than in the control group. In addition, both groups had increased plasma IL-6 levels compared to the levels of the same marker obtained before surgery [59]. TNF-α levels also did not show significant changes before surgery, but 3 h after surgery, there was a considerable increase in TNF-α levels in non-obese individuals when compared to obese individuals [59].

Adipose tissue can have distinct effects on the inflammatory process. This is due to the fact that, adipose tissue contains two classical macrophage phenotypes: pro-inflammatory (M1) and anti-inflammatory (M2) [60]. In non-obese mice, CD206 and CD163 receptors are expressed in the M2 macrophages [61], whereas4 obesity induces a prominent population of macrophages in the adipose tissue expressing CD11, CD9, and TREM2 (triggering receptor expressed on myeloid cells 2) [62]. Regarding inflammatory mediators, a study evaluated the behavior of the CRP in visceral and subcutaneous adipose tissues and its connection with two inflammatory markers, IL-6 and TNF-α [63]. Six months following Roux-en-Y gastric bypass intervention, CRP showed a 50% decrease when compared to the control group, and 1 year after surgery, the levels were even lower [56]. Between the two inflammatory markers, only IL-6 showed a positive association with CRP [63]. When individuals with dyslipidemia were evaluated, there was a 50% increase in CRP concentration, which may indicate an association with cardiovascular disease [63]. Another study evaluated visceral adipose tissue inflammation through the gene expression of systemic inflammatory markers in obese individuals after sleeve gastrectomy or even gastric bypass [64]. The study revealed a positive association between circulating levels of leptin, hs-CRP, and BMI. Additionally, gene expression of the pro-inflammatory cytokine leptin changed according to the participants’ body mass index. It is important to stress that the magnitude of weight loss achieved through the surgical procedure had a positive relationship with the visceral inflammatory status of the study participants [64].

Interestingly, several studies have compared the inflammatory responses induced by the two weight loss methods: weight loss through diet and surgery. A study evaluated two groups: obese women who lost weight through a diet program and through Swedish adjustable gastric band bariatric surgery [65]. The results showed that the levels of TNF-α were increased in individuals who underwent surgery when compared with the diet group alone; however, there was a decrease in insulin resistance in the same group [65]. An earlier study has also revealed the impact of bariatric surgery on some inflammatory mediators, indicating that TNF-α remains unaltered despite weight loss [66]. Such results should be interpreted on the basis of specific eating behaviors as well as weight control practices as a result of weight loss.

## 5. DNA Damage

Some studies have demonstrated the association between obesity and ROS as well as between ROS and genetic alterations [67,68]. Obesity creates an environment that promotes severe oxidative stress by impairing ROS production and antioxidant defenses, leading to DNA strand damage (strand breaks, adducts, and DNA cross-links). Metabolic dysregulation and inflammation are associated with obesity and induce DNA damage by releasing pro-inflammatory cytokines as well as activating immune cells [69]. Additionally, genetic damage is established to be responsible for genomic instability, which is a pivotal event in the development of many chronic diseases, including cancer [70,71]. Higher frequencies of mutations were observed in the erythrocytes of obese mice than in non-obese mice [72]. A previous study by our research group demonstrated genetic damage in the liver cells of obese Zucker rats using single-cell gel comet assays [73]. Some studies have shown that DNA damage in the kidney, liver, and colon cells of obese Zucker rats can be alleviated by gastric bypass surgery or even chronic caloric restriction [74]. Studies conducted by Bankoglu et al. [75] have also found a decrease in the number of micronucleated cells and primary DNA damage in peripheral lymphocytes from obese individuals 12 months but not 6 months after bypass surgery. The proliferation index and mitosis were increased, followed by a significant reduction in apoptosis, in these volunteers. According to the authors, since the changes could be detected only after 12 months, the significant reduction in body weight for long periods plays an important role in reducing genotoxicity [76]. This may be due to the fact that an important reduction in oxidative stress after bariatric surgery-induced weight loss has been detected [77,78]. Low expression of pro-inflammatory and oxidative stress markers was observed 1 year after surgery, followed by daily vitamin supplementation, although additional antioxidant supplementation was necessary [79].

Epigenetics is the genome-environment interaction that can have an effect on gene expression following development and aging. Some authors have postulated that metabolic diseases are strongly associated with epigenetic changes, assuming that epigenetic factors play a crucial role in obesity [80]. In fact, altered gene expression has also been observed in obese individuals [81,82,83]. However, the studies show that Roux-en-Y gastric bypass-induced weight loss has a dynamic effect on the epigenome, with epigenetic regulation being a relevant biological pathway in the expression of genes closely involved either in the physiology or metabolism of human obesity [84,85,86,87]. Thus, structural changes in clinical chemistry following weight-loss surgery may influence DNA methylation and subsequent mRNA expression. Among the epigenetically controlled genes in skeletal muscle, the DNA methylation patterns of PGC1A (peroxisome proliferator-activated receptor-gamma coactivator-1alpha) and PDK4 (pyruvate dehydrogenase kinase 4) showed significant alterations [88]. PGC1A is a transcriptional coactivator that plays a pivotal role in the modulation of cellular energy metabolism as a result of the stimulation of mitochondrial biogenesis. It is highly expressed in many tissues where mitochondria are present as long as oxidative metabolism is always active, such as brown adipose tissue, skeletal muscle, and the heart [88]. PDK4 encodes a mitochondrial protein that regulates glucose metabolism. An early study showed an association between increased ROS production and enhanced PDK4 expression in mouse white adipose tissue [89]. Although weight loss caused by bariatric surgery may reduce cancer risk in obese patients by reducing mutations, PDK4 modulation can stimulate cell proliferation and tumorigenesis [90]. Overexpression of PDK4 has been detected in gastric cancer cells, demonstrating a correlation with clinicopathological characteristics and a poor prognosis [91]. Methylation and subsequent modulation of specific genes belonging to inflammatory pathways have also been found after surgery [92]. Methylation increases were observed in promoters of PDK4, IL-B, IL-6, and TNF-α genes. In general, the increased methylation in promoters of the genes of these inflammatory mediators will enhance cancer development, although this may be considered a premature conclusion that needs further studies [93]. However, depending on the type of obesity treatment (diet or even surgery), DNA methylation may vary considerably. Roux-en-Y gastric bypass induced more profound epigenetic changes than did a very low-calorie diet in the promoters of the genes for TFAM, IL1 B, PARGC1 A, IL6, and TNF genes [94]. More recently, a study clarified that promoter methylation of the NFKB1 gene increased after bariatric surgery, being closely associated with circulating levels of some inflammatory markers and decreased blood pressure [94].

In summary, a less obvious but equally important aspect of obesity has been revealed by recent scientific discoveries: its profound impact on DNA integrity and stability.

## 6. Apoptosis

Several studies have investigated the cytotoxic effects of gastric bypass surgery in order to better understand the biological mechanisms by which gastric bypass surgery modulates the cellular machinery closely related to death. Apoptosis is a conserved biological, genetically controlled cell death process for normal development and subsequent tissue homeostasis [95]. Some studies have evaluated the idea that obesity can induce apoptosis in the peripheral blood of overweight individuals [75]. Furthermore, in obesity, a higher degree of insulin resistance causes apoptosis of liver cells. During apoptosis, there is the activation of caspases capable of cleaving various substrates, such as cytokeratin-18, the most important intermediate filament protein found in the liver. The authors suggest that inflammation and insulin resistance are important factors in the induction of hepatocyte apoptosis and steatohepatitis, rather than the degree or severity of obesity [96]. Most importantly, the incidence of apoptotic cells pointed out statistically significant differences between normal-weight and total overweight/obese individuals [75].

The TUNEL assay showed severe myocardial cell apoptosis in the sham-operated control group compared to that in the duodenal–jejunal bypass and sleeve gastrectomy groups [97]. In contrast, bariatric surgery substantially decreased the mRNA expression levels of PERK, CHOP, GRP78, and caspase 12, indicating that the intervention reduced cardiomyocyte endoplasmic reticulum (ER) stress [97].

Moreover, bariatric surgery prevents the onset of Alzheimer’s disease by increasing glucagon-likepeptide-1 (GLP-1) levels [98]. Growing evidence has demonstrated that GLP-1 comprises a class of antidiabetic drugs able to promote weight loss for obese individuals with or without diabetes [99]. These medicines act by binding to the GLP-1 receptors in many organs, such as the intestines, pancreas, and central nervous system, in order to enhance downstream metabolic pathways [100]. In particular, GLP-1 increases insulin secretion and pancreatic islet neogenesis, induces satiety, inhibits apoptosis, and regulates blood pressure and cardiac function [93]. Roux-en-Y gastric bypass can bring subcutaneous adipose tissue into a less pathological state and cause lower stress, as fewer inflammatory processes were observed [101]. Moreover, Roux-en-Y gastric bypass surgery activates unacylated ghrelin, plasma acylated ghrelin, and obestatin levels, indicating that the ghrelin system has a key role in the regulation of intracellular calcium mobilization and the reduction in apoptosis in the pancreas. Ghrelin gene expression protects β-cells by regulating calcium homeostasis [102]. Ghrelin therapy reduced TNF-α-induced apoptosis, as evidenced by lower caspase-8 and -3 cleavage in vitro [103]. As a result, a severe reduction in autophagic flux was detected in obese adipocytes when compared to the controls, which was inversely correlated with fat cell lipids.

Overall, these findings suggest two important observations that deserve attention: bariatric surgery prevents cell death, and karyolysis is closely associated with necrosis [89]. Further studies are required to better understand the effect of bariatric surgery on cell death.

## 7. Concluding Remarks

When weight loss was achieved by diet, medication, and/or surgery, the results were compared with the preferences that patients express when entering a weight loss program. Only in bariatric surgery had enough weight loss been achieved to make most people with obesity happy with their results [3]. Bariatric surgery is undoubtedly a relevant and important option for obese patients because obesity is a serious public health problem in the vast majority of countries in the world today. However, clarifying whether there is a risk of cancer in these individuals through the study of biological markers related to oxidative stress is of utmost importance, particularly because there is little information in the literature to date.

This review suggests that obesity-related abnormalities may be alleviated by bariatric surgery. In particular, it may inhibit reactive oxygen species synthesis, improve the antioxidant system, prevent apoptosis in the peripheral blood, hepatic cells, and myocardial cells, and modulate inflammation. These recent findings are summarized in Table 1. Moreover, the reduction in DNA damage strongly depends on the maintenance of a low body weight for long periods of time. However, the data in the literature are not yet sufficient to confirm the association between bariatric outcomes and inflammation, apoptosis, DNA damage, oxidative stress, and tumor formation. Certainly, a better understanding of the effects will help improve its impact on obese patients, in particular the adoption of therapeutic strategies for mitigating the undesirable outcomes induced by the surgical procedure.

## Figures and Tables

**Table 1:** The main findings in the last five years from studies investigating oxidative stress responses in patients submitted to bariatric surgery.

Author(s)	Casuistics (n)	Surgical Procedure	Time of Evaluation	Main Biochemical and Molecular Findings after Bariatric Surgery
Perri et al. [35]	23	Sleeve-gastrectomy	3 months after surgery	↓ Oxidative stress, biochemical markers, body composition parameters
Carmona-Maurici et al. [38]	24	Roux-en-Y gastric bypass or sleeve-gastrectomy	12 months after surgery	↓ levels of oxidized low-density lipoprotein, lipoprotein (a) and superoxide dismutase-2 levels. ↑ antioxidant enzymes, specifically paraoxonase-1 and catalase.
Min et al. [39]	19	Sleeve gastrectomy, biliopancreatic diversion, Roux-en-Y gastric bypass, and laparoscopic adjustable gastric banding	1, 6 and 48 months after surgery	↓ C-reactive protein, interleukin-6, and leptin. ↑ antioxidant status
Choromańska et al. [42]	40	Laparoscopic adjustable gastric band	36 months after surgery	↓ hyperglycemia, A1C levels ↑ HDL-cholesterol levels
Choromańska et al. [43]	65	Sleeve gastrectomy.	1, 3, 6, and 12 months after surgery	↓ Superoxide dismutase and reduced glutathione (GSH), and uric acid levels ↑ Glutathione reductase, oxidative damage to proteins (advanced glycation end products, AGE; advanced oxidation protein products, AOPP) and lipids (8-isoprostanes, 8-isop; 4-hydroxynonenal).
Fariello et al. [44]	15	Roux-en-Y gastric bypass	12 months after surgery	↓ lipid peroxidation, estradiol levels ↑ higher levels of luteinizing hormone (LH), sex hormone-binding globulin (SHBG), and testosterone.
Carlsson et al. [46]	356	Roux-en-Y gastric bypass surgery	12 months after surgery	↑ 8-oxodG ↓ 8-oxoGuo, hyperglycemia and insulin resistance.
Picu et al. [50]	41	Sleeve gastrectomy	6 and 12 months after surgery	↓ glycated hemoglobin (HbA1c), blood glucose levels, BMI, weight, visceral fat level, HDL-cholesterol, incretin hormones, pro-inflammatory markers, and oxidative stress
Metere et al. [51]	20	Sleeve gastrectomy	1, 3, 6, and 12 months after surgery	↓ Oxidative stress (OH, O_2_^•^, ONOO^−^, and NO).
Katsogiannos et al. [55]	34 ng	Roux-en-Y gastric bypass (RYGB)	6 months after surgery	↓ glycated hemoglobin A_1c_.
Jouan et al. [58]	87	Gastric bypasses, or sleeve gastrectomy	12 months after surgery	↓ Pro-inflammatory markers (IL-6, CRP, leptin, resistin) ↑ anti-inflammatory markers (IL-10, adiponectin)
Bankoglu et al. [74]	56	Not informed	6 and 12 months after surgery	↓ DNA damage, ferric-reducing antioxidant power assay ↑ level of oxidized glutathione and lipid peroxidation products at 6 months but normalized at 12 months after surgery.
Fraszczyk et al. [84]	40	Roux and Y Gastric Bypass	3, 6, and 12 months after surgery.	↓ fasting glucose, HbA1c, HOMA-IR, insulin, total cholesterol, triglycerides, LDL and free fatty acids levels. ↑ HDL levels
		Lap		
		Biliopancreatic diversion		
		Gastric sleeve		
Ezquerro et al. [103]	30	Roux-en-Y gastric bypass	6 months after surgery	↓ Desacyl ghrelin and, acylated/desacyl ghrelin levels

↑ increased ↓ decreased.

## Data Availability

The data are contained within the article.
